# HyPRP1 Gene Suppressed by Multiple Stresses Plays a Negative Role in Abiotic Stress Tolerance in Tomato

**DOI:** 10.3389/fpls.2016.00967

**Published:** 2016-06-29

**Authors:** Jinhua Li, Bo Ouyang, Taotao Wang, Zhidan Luo, Changxian Yang, Hanxia Li, Wei Sima, Junhong Zhang, Zhibiao Ye

**Affiliations:** ^1^Key Laboratory of Horticulture Science for Southern Mountainous Regions, Ministry of Education; College of Horticulture and Landscape Architecture, Southwest UniversityChongqing, China; ^2^Key Laboratory of Horticultural Plant Biology (MOE), Huazhong Agricultural UniversityWuhan, China

**Keywords:** abiotic stress, oxidative stress, drought stress, hybrid proline-rich protein, tomato

## Abstract

Many hybrid proline-rich protein (HyPRP) genes respond to biotic and abiotic stresses in plants, but little is known about their roles other than as putative cell-wall structural proteins. A *HyPRP1* gene encodes a protein with proline-rich domain, and an eight-cysteine motif was identified from our previous microarray experiments on drought-tolerant tomato. In this study, the expression of the *HyPRP1* gene in tomato was suppressed under various abiotic stresses, such as drought, high salinity, cold, heat, and oxidative stress. Transgenic functional analysis showed no obvious changes in phenotypes, but enhanced tolerance to various abiotic stresses (e.g., oxidative stress, dehydration, and salinity) was observed in RNAi transgenic plants. Interestingly, several SO_2_ detoxification-related enzymes, including sulfite oxidase, ferredoxins (Fds), and methionine sulfoxide reductase A (Msr A), were revealed in HyPRP1-interacting proteins identified by Yeast Two-Hybrid screening. More sulfates and transcripts of *Msr A* and *Fds* were accumulated in *HyPRP1* knockdown lines when wild-type plants were exposed to SO_2_ gas. Our findings illustrate that the tomato *HyPRP1* is a negative regulator of salt and oxidative stresses and is probably involved in sulfite metabolism.

## Introduction

Hybrid proline-rich proteins (HyPRPs) comprise a dynamically evolving protein family unique to seed plants (Dvorakova et al., 2007) and are initially defined as proteins that respond to wounding (Chen and Varner, 1985). HyPRPs are putative cell-wall proteins consisting of a repetitive proline-rich N-terminal domain and a conserved eight-cysteine motif (8CM) C-terminal domain. Thus, HyPRPs belong to the 8CM superfamily, which also contains protease inhibitors, lipid-transfer proteins, and several other protein subgroups (Jose-Estanyol et al., 2004; Dvorakova et al., 2007).

HyPRPs have been widely reported to respond to biotic and abiotic stresses. A HyPRP1gene in *Capsicum annuum* and *Nicotiana benthamiana* performs dual roles in the positive regulation of cell death and negative regulation of basal defense against pathogens (Yeom et al., 2012). A heterologously expressed *Arabidopsis* HyPRP gene *EARLI1* can improve the survival of yeast cells in freezing conditions (Zhang and Schlappi, 2007); A pigeon pea HyPRP gene (CcHyPRP) expressed in yeast and *Arabidopsis* affords multiple abiotic stress tolerance (Priyanka et al., 2010). Similarly, the *EARLI1* in *Arabidopsis* was found that it plays an auxiliary role for low temperature and salt stress protection responses (Xu et al., 2011), and the overexpression of *Medicago falcata* HyPRP (MfHyPRP) in tobacco increased its tolerance to freezing, chilling, osmotic stress, and methyl viologen (MV)-induced oxidative stress (Tan et al., 2013). However, little is known about the functional roles of HyPRP and its molecular mechanism in abiotic stresses in tomato.

Abiotic stresses such as drought, salinity, and extreme temperature are major factors inhibiting the growth, development, and productivity of crops (Hou et al., 2009; Budak et al., 2015). In agriculture, these abiotic stresses can become overwhelming with global climate changes and directly cause extensive losses in crop production and quality worldwide (Mittler, 2006; Spicher et al., 2016). Understanding the response mechanisms of plants to these abiotic stresses is an important field in plant research (Hirayama and Shinozaki, 2010). Most abiotic stresses directly or indirectly lead to rapid accumulation of toxic products, such as free radicals and reactive oxygen species (ROS), which cause oxidative stress (Oberschall et al., 2000). Any protection against abiotic stress is believed to be caused by the direct or indirect scavenging of ROS (Vickers et al., 2009).

The antioxidant machinery is sufficient to maintain equilibrium between production and scavenging of ROS under normal physiological conditions, and such balance is commonly known as redox homeostasis. However, the static lifestyle of plants causes them to be interminably exposed to unfavorable environmental conditions, such as extreme temperatures, high light intensities, drought, salinity, air pollution, and pathogen attack, all of which are known to increase the rate of ROS generation (Spicher et al., 2016). When ROS production overwhelms the cellular scavenging capacity that suspends cellular redox homeostasis, the result is a rapid and transient excess of ROS, known as oxidative stress (Scandalios, 1997). Unlike ROS, SO_2_ is an external source of toxic stimuli for plants and is known as a damaging air pollutant that can be transformed into sulfite, the main component of acid rain (Lang et al., 2007).

Rapid climate changes caused by human activities pose a serious threat to biodiversity and the ecosystem. Although species have adapted to environmental changes for millions of years, rapid climate change requires larger scale and faster adaptation than before (http://www.epa.gov). Although, cultivated tomato (*Solanum lycopersicum*) is sensitive to drought and salt (Gong et al., 2010), a wild tomato species (*S. pennellii*) shows strong adaptation to arid environments owing to its high water-use efficiency (Martin and Thorstenson, 1988) and the ability of its leaves to absorb dew (Rick, 1973). To explore the drought-resistant mechanism of *S. pennellii*, a drought-suppressed *HyPRP1* gene was screened out using an oligonucleotide microarray in our previous research (Gong et al., 2010). In the present study, we found that the expression of *HyPRP1* is suppressed by various abiotic stresses, including drought, high salinity, cold, heat, oxidative stress, and phytohormone ABA in *S. pennellii*. *SlHyPRP1* and *SpHyPRP1* were isolated from cultivated tomato *S. lycopersicum* cv. M82 and wild tomato *S. pennellii* LA0716, respectively, and encode different structural proteins, as well as play different roles in ROS tolerance in *Escherichia coli* cells. Transgenetic functional analysis and transcriptional investigation demonstrated that *HyPRP1* possibly plays a negative role in stress tolerance.

## Materials and methods

### Plant materials and stress treatments

Tomato plants (*S. pennellii* LA0716) were grown in a naturally illuminated glasshouse. Tissues from the roots, stems, leaves, flowers, and fruits at various developmental stages were collected from untreated control plants, immediately frozen in liquid nitrogen, and stored at −80°C. For gene expression profiling analysis, identical 2-month-old tomato plants were subjected to various stresses or plant growth regulator treatments. Salt, drought, cold, heat, wounding, ABA treatments, and oxidative stress were simulated as previously described (Loukehaich et al., 2012). Briefly, salt stress was simulated by watering plants with 200 mM NaCl solution, and drought stress was simulated by placing detached leaves on filter paper under 70% relative humidity at 25°C. Cold and heat was imposed by transferring plants to a growth chamber and holding the plants at 4 or 40°C, respectively. Wounding was performed by pinching the leaves with forceps. For ABA treatments and oxidative stress, tomato plants leaves were directly sprayed with 100 μM ABA and 100 μM MV until run-off. Three leaves from various treated and untreated plants were collected at different time points and stored as described above.

### RT-PCR and qRT-PCR

Total RNA was isolated using TRIzol reagent (Introvigen, USA). Dnase I (Fermentas, USA) treated RNA is reverse-transcribed using ReverTra Ace reverse transcriptase (TOYOBO, Osaka, Japan). Real-time quantitative RT-PCR (qRT-PCR) was performed on a LightCycler Roche 480 (Roche Diagnostics, Basel, Switzerland) with a LightCycler 480 SYBR Green I Master kit (Roche) used in accordance with the supplier's instructions. The PCR amplification consisted of an initial incubation at 95°C for 5 min, followed by 40 cycles for 10 s at 95°C, 15 s at 58°C, and 20 s at 72°C. Data were gathered during the extension step. Melting-curve acquisition and analyses were also performed on the cycler. Each sample included three replicates, and the data were normalized against the reference β*-actin* gene (Solyc11g005330.1.1). The three replicates in qRT-PCR assay were three technical replicas, each assayed sample represents three independently collected samples. The qRT-PCR assays are from one of two different experiments that yielded essentially identical results. The expression of tomato oxidative related gene *SlCAT* (Solyc12g094620.1.1), *SlSOD* (Solyc09g082690.2.1), and *SlMSR B* (EF144171) was analyzed by real-time PCR in wild-type (WT) and transgenic plants. The qRT-PCR primer sequences are listed in Table 1S.

### Vector construction and transgenic analysis

A full length of *SpHyPRP1* (Sopen12g004640.1, SGN: https://solgenomics.net/) cDNA from *S. pennellii* LA0716 was amplified with the forward primer 5′-CAATCTTTGTACCAA ATTATTTAACCA-3′ and reverse primer 5′- AACAATT CCACAAAGCCAAAA-3′. The PCR product was cloned into the pMD18-T vector (TaKaRa, Dalian, China) and then sequenced. pMD18-T-*SpHyPRP1* was digested with restriction enzymes *SalI* and *KpnI*. The resulting product was inserted into the *XhoI* and *KpnI* sites of the binary vector pMV (pBI121 reformed) to yield the overexpressing construct with *SpHyPRP1*, which was driven by a cauliflower mosaic virus 35S promoter (CaMV35S). To construct the RNA interference vector, a 451 bp fragment was amplified from the *SlHyPRP1* coding sequence (Solyc12g009650.1.1) by using gene-specific primers with a 5′-attB1 extension forward primer GGGGACAAGTTTGTACAAAAAAGCAGGCTTCTTTGTACCAAATTATTTAACCA CA and a 5′-attB2 extension reverse primer GGGGACCACTTTGTACAAGAAAGCTGGGTCAATTGGTGGAACTGT GACC (5′-attB1 and 5′-attB2 extensions are underlined). A recombination reaction between the PCR product and the pHellsgate 2 vector (Invitrogen, USA) was performed using BP clonase (Invitrogen) in accordance with the manufacturer's instructions. Both constructs were used to transform tomato *S. lycopersicum* M82 mediated by the *Agrobacterium tumefaciens* strain C58. A copy of the T_0_ transgenic tomato plant was detected through Southern blot hybridization by using neomycin phosphotransferase II gene as the probe. The expression of *HyPRP1* in *HyPRP1*-RNAi and overexpressed transgenic (T_0_, T_1_, and T_2_) plants was examined by qRT-PCR as described above. The transgenic T_2_ or T_3_ lines with *HyPRP1* transcripts that increased or decreased significantly were used for further analysis.

### Abiotic stress assays

Positive transgenic seedlings from three lines were germinated in 1/2 MS medium for 2 days and then subcultured in 1/2 MS containing 150 mM NaCl, 200 mM mannitol, or 3 μM ABA. The seedlings were grown for 12 days, each with three replicates.

To evaluate the salt tolerance of transgenic lines, uniform-sized positive seedlings that were confirmed through PCR were transplanted into cylindrical pots (diameter: 8 cm, height: 15 cm) and grown up to the five-leaf stage. Afterward, the seedlings were treated with either 75 μM MV with 1 mg/L Tween-20 sprayed on the leaves until run off or 200 mM NaCl by watering the plants, each with three to four replicates. The relative seedling growth and root weight were measured by dividing the treated seedling height and root weight by those of the untreated plants and multiplying the result by 100 (Hou et al., 2009).

To determine the water loss rate, 15 leaves were collected from the same location in transgenic and WT plants. The weights of the leaves were measured progressively at specified time points. After 3 h of dehydration, the accumulation of H_2_O_2_ in the leaves detached *in situ* was examined by histochemical staining with 3, 3′-diaminobenzidine (DAB). Briefly, all the leaves were stained with DAB solution (1 mg/mL) and incubated for 4 h at 25°C in the dark. Samples were then cleaned with 70% alcohol and incubated at 70°C for 10 min.

### SO_2_ treatment and sulfate content

Plants at the six-leaf stage were subjected to SO_2_ stress treatment. SO_2_ exposure was carried out in a transparent 1 m^3^ growth chamber. The corresponding weight (2 or 5 g) of sublimed sulfur was burned in the chamber to produce ~4 or 10 ppm SO_2_. The chambers were sealed with transparent adhesive tape and gently shaken to keep the SO_2_ evenly distributed inside. Both the control (plants in a chamber without SO_2_ treatment) and treated plants were kept under continuous light at 25°C with ~85 to 95% relative humidity. To determine the inner sulfate level, *HyPRP1* knockdown, overexpression lines, and WT control plants were exposed to 10 ppm SO_2_ for 2 h. The leaves were then cut and extracted immediately in double-distilled water, followed by heating for 5 min at 95°C (Hansch et al., 2006). Sulfate content was determined using an ICS-1000 ion chromatography system (Dionex, USA) equipped with an electrochemical conductivity detector (DS6, Dionex) combined with an upstream-inserted micromembrane suppressor (ASRS-Ultra II 4 mm, Dionex) and a Dionex IonPac AS9-HC column, which was used to separate the mobile phase containing 9.0 mM Na_2_CO_3_ at a flow rate of 1.5 mL/min.

### Chlorophyll content assay

The chlorophyll content was measured by Lichtenthaler method (Lichtenthaler, 1987). Leaf tissues were ground under liquid nitrogen and extracted with 8 mL of 95% (v/v) ethyl alcohol. Absorption spectra were detected at 665 and 649 nm. Chlorophyll was computed using the following equation: chlorophyll concentration (mg/mL) = (6.63 × A665) + (18.08 × A649), where A is the absorbance at a specified wavelength.

### Yeast two-hybrid screening and assay

For the yeast two-hybrid (Y2H) screenings, the full coding sequence of *SpHyPRP1* was cloned by PCR following the amplification of the cDNA sequence by using the forward primer 5′-CCCGGGAATGGAGTTCTC TAAGATAACTTCA-3′ and the reverse primer 5′-CTGCAGCTAG ATGGAACAAGTGTAGCCAG-3′. The PCR fragment was cloned into the pMD18-T vector (TaKaRa, Dalian, China) and confirmed by sequencing. The correct plasmid was digested with *SmaI* and *PstI*, and the fragment was fused to the frame with the GAL4 DNA-binding domain into the *SmaI* and *PstI*-digested pGBKT7 vectors. The bait construct pGBKT7-SpHyPRP1 was transformed into the yeast strain Y187 through lithium acetate method. Interacting clones were screened through mating in accordance with the manufacturer's instructions (Clontech, USA), and 60 randomly selected positive clones were sequenced and analyzed.

### Bimolecular fluorescence complementation (BiFC) analysis

The full-length cDNA of *SpHyPRP1* without the stop codon was amplified using PCR and cloned into the N-terminal 155 amino acid portion of yellow fluorescent protein (YFPN) in the pUC-SPYNE^G^ vector (Walter et al., 2004) to induce SpHyPRP1::YFP^N^ fusion. Full-length cDNAs of Msr A, UBQ10, Fds, ZPR1, and SO without the stop codon were also amplified through PCR by using a pair of primers (Table 1S). The fragment was fused into the C-terminal 84 amino acid portion of YFP (YFPC) in the pUC-SPYCE^G^ vector to generate Msr A::SPYCE^G^, SO::SPYCE^G^, Fds::SPYCE^G^, ZPR1::SPYCE^G^, and UBQ10::SPYCE^G^ fusion proteins. The corresponding constructs were co-delivered by bombarding the gold-coated vectors into tobacco BY-2 (*N. tabacum* cv. Bright Yellow 2) cells by using Biolistic PDS-1000 (Bio-Rad, USA). All samples were observed under a Leica TCSST2 confocal laser microscope (Zeiss, LSM510, Germany) after 24 h of bombardment.

### SpHyPRP1 and SlHyPRP1 expression in *E. coli*

The full-length ORF of *SpHyPRP1* and *SlHyPRP1* was amplified through PCR by using the forward primer 5′-GG ATCCATGGAGTTCTCTAAGATAACTTCA C-3′ and the reverse primer 5′-CTCGAGCTAGATGG AACAAGTGTAGCCAG-3′ from *S. lycopersicum* cv. M82 and *S. pennellii* LA0716, correspondingly. The amplicons were inserted into the pEASY-E1 vector (TransGen Biotech, China) through TA cloning, in which the exogenous gene was under the control of a T7 RNA polymerase promoter. The resulting constructs pET-SlHyPRP1 and pET-SpHyPRP1, together with the empty vector pEASY-E1, were introduced into *E. coli* BL21 (DE3) cells. The correct clones confirmed by sequencing were used for further analysis. To measure the growth rate under oxidative stress, *E. coil* cells with either of the above plasmids were grown in LB liquid media containing 100 μg/mL ampicillin with continuous shaking at 37°C. When the cells were grown to an absorbance value of A_600nm_ = 0.6–0.8, 1 mM isopropyl-1-thio-β-galactopyranoside was used to induce gene expression for 4 h. Subsequently, the cells were diluted 10 times using new LB media with antibiotics and grown further to the mid-log phase (A_600nm_ = 0.3–0.6). The cells were then challenged with 1.5 mM H_2_O_2_. The attenuance at 600 nm was measured at designated time points. Afterward, 2 μL of cells were also dotted into the LB agar plates supplemented with 1.5 mM H_2_O_2_. Colony formation was observed after 4 h of incubation at 37°C.

## Results

### Isolation and characterization of *HyPRP1* in tomato

**Figure 1:**
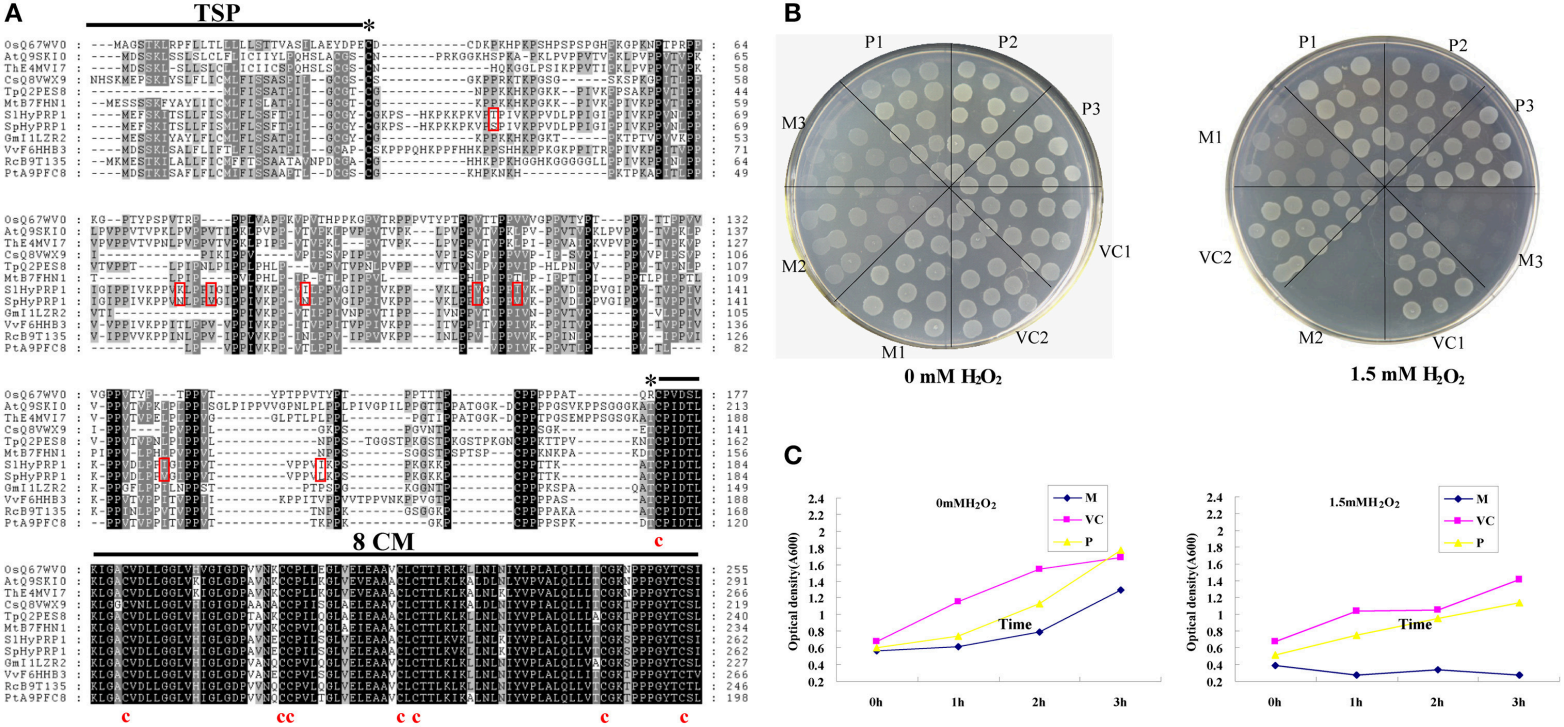
**Comparison of amino acid sequence between SlHyPRP1 and SpHyPRP1. (A)** Amino acid alignment of *S. lycopersicum* cv. M82 (SlHyPRP1) and *S. pennellii* LA0716 (SpHyPRP1); and the HyPRP1 sequences of *Vitis vinifera* (VvF6HHB3), *Ricinus communis* (RcB9T135), *Glycine max* (GmI1LZR2), *Arabidopsis thaliana* (AtQ9SKI0), *Thellungiella halophila* (ThE4MVI7), *Trifolium pratense* (TpQ2PES8), *Populus trichocarpa* (PtA9PFC8), *Medicago truncatula* (MtB7FHN1), *Cucumis sativus* (CsQ8VWX9), and *Oryza sativa* (OsQ67WV0). Eight conserved cysteines in the 8CM are indicated below the alignment. The asterisks indicate the beginning and end of the proline-rich repetitive domain (PRD). The transmembrane signal peptide (TSP) of SpHyPRP1 and SlHyPRP1 is indicated below the alignment by the SOSUI program (http://bp.nuap.nagoya-u.ac.jp/sosui/). The red box indicates single-amino-acid differences between SpHyPRP1 and SlHyPRP1. **(B)** Growth response of *E. coli* expressing *SlHyPRP1 or SpHyPRP1* under oxidative stress conditions. Colony formation of *E. coli* strains on the LB plates, supplemented with and without 1.5 mM H_2_O_2_, from one of three different experiments that yielded essentially identical results. **(C)** Growth rate of *E. coli* strains in the presence of 1.5 mM H_2_O_2_. Values are shown for one representative of three independent experiments. M, P, and VC are the *E. coli* cells transformed with pET-*SlHyPRP1*, pET-*SpHyPRP1* plasmid, and empty pET-E1 vector, respectively. Serial numbers 1, 2, and 3 are different clones of transformed *E. coli*.

In our previous studies on drought stress in tomato introgression lines (ILs), a differential expression profile of the *SlHyPRP1* gene was observed between the drought-tolerant ILs and M82 (Gong et al., 2010). To clarify the function of HyPRP1 in abiotic stress, the full-length cDNAs of SlHyPRP1 and SpHyPRP1 were isolated from *S. lycopersicum* cv. M82 and *S*. *pennellii* LA0716 by RT-PCR, respectively. Both *SlHyPRP1* and *SpHyPRP1* encoded 262 amino acids predicted by the FGENESH program (http://linux1.softberry.com/berry.phtml). These two putative amino acids shared 96% similarity, differing only in eight residues. Threonine at site 43 (T) and isoleucine 85 (I), 120 (I), and 150 (I) in *S. lycopersicum* cv. M82 were substituted by serine 43 (S) and valine 85 (V), 120 (V), and 150 (V) in *S*. *pennellii* LA0716, correspondingly. These findings suggest that a major amino acid difference between the two species is the demethylation in *S*. *pennellii* LA0716 at a corresponding site. One exception was observed in site 115, where the V in *S. lycopersicum* cv. M82 was methylated to I. At sites 81 and 95, the positively charged residue lysine (K) in M82 was replaced by asparagine (N) with neutral residue in LA0716. The most prominent feature was found at site 95, where the hydrophobic isoleucine (I) in M82 was substituted by hydrophilic asparagine (N) in LA0716 (Figure 1A).

A similar search of the GenBank database revealed that HyPRP1 in tomato shares a significant degree of sequence identity at 8CM residues with other HyPRPs from various species of plants. In addition, the proline-rich repetitive domain (PRD) at the N-terminus showed a varied repeated order and high proline content (Figure 1A). According to the prediction results of the SOSUI program (http://bp.nuap.nagoya-u.ac.jp/sosui/), SlHyPRP1 and SpHyPRP1 exhibited one putative transmembrane signal peptide and an average hydrophobicity of 0.52 and 0.48, respectively. This finding implies that HyPRP1 is a transmembrane protein, which may differ in hydrophobicity between *S. lycopersicum* and *S*. *pennellii*.

By using plant cDNAs expressed in *E. coli*, transformants enhanced the host abiotic stress (Garay-Arroyo et al., 2000; Mundree et al., 2000; Yamada et al., 2002; Shin et al., 2008). Therefore, to further examine the functional differences between *SlHyPRP1* and *SpHyPRP1*, the prokaryotic expression of these two proteins was carried out, and the tolerance of the host *E. coli* cells to oxidative stress was evaluated. Moreover, *E. coli* cells that expressed *SlHyPRP1* exhibited noticeably reduced resistance to ROS-inducing reagents in both solid and liquid media. By contrast, the cells that expressed *SpHyPRP1* only showed slightly reduced oxidative tolerance (Figures 1B,C). These results indicated that *HyPRP1* protein plays a negative role in scavenging ROS in *E. coli* and exhibits different effects based on the introduction of *SlHyPRP1* or *SpHyPRP1*.

### Transgenic lines overexpressing *SpHyPRP1* are sensitive to salt, mannitol stress, and ABA

**Figure 2:**
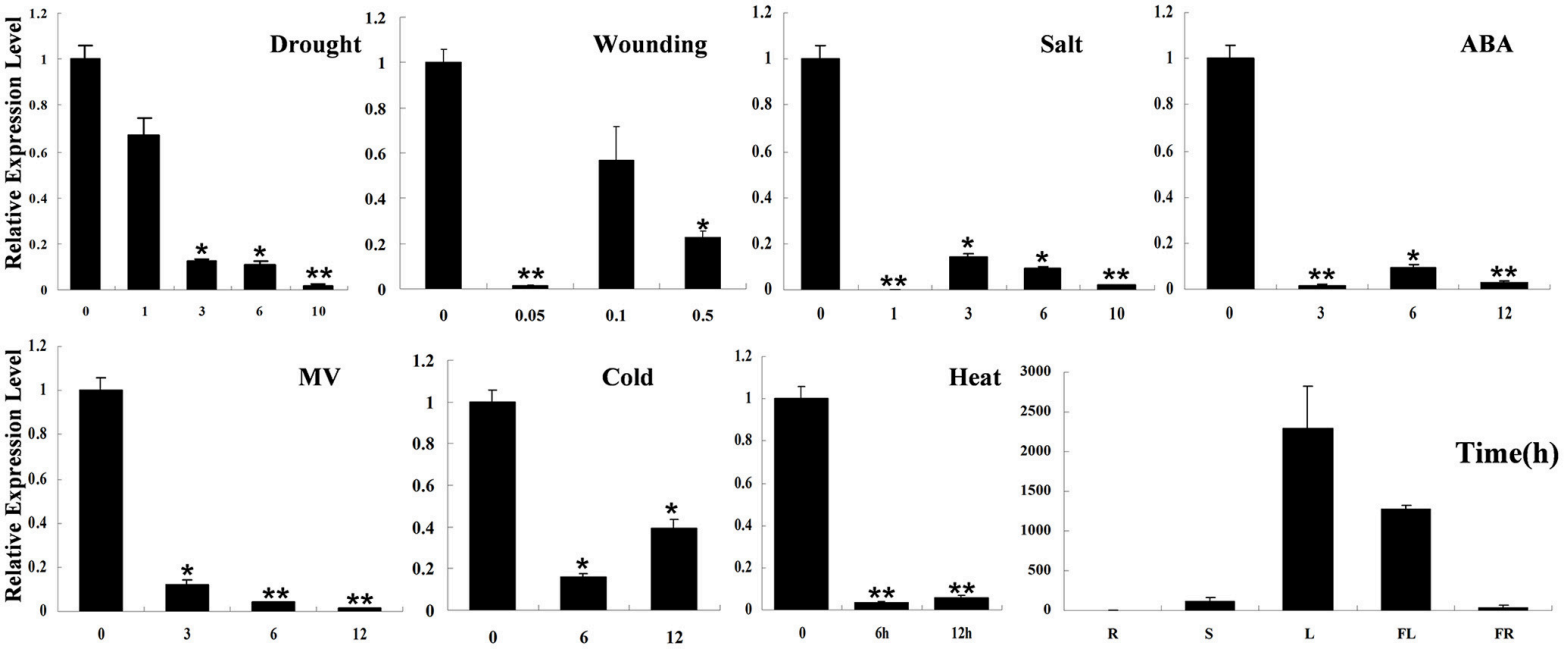
**Expression profiles of *HyPRP1* in different tissues (R, root; S, stem; L, leaf; FL, flower; FR, fruit) and in the leaf of *S. pennellii LA0716* under various stresses (e.g., drought, salt, heat, cold, and MV) and ABA treatments**. All samples were collected at the indicated time points from three biological replicates of each treatment. Single (*P < 0.05) and double (**P < 0.01) asterisks denote statistically significant differences between the stress treatment and the 0 h control. Error bars indicate ± SE of the means (*n* = 3).

**Figure 3:**
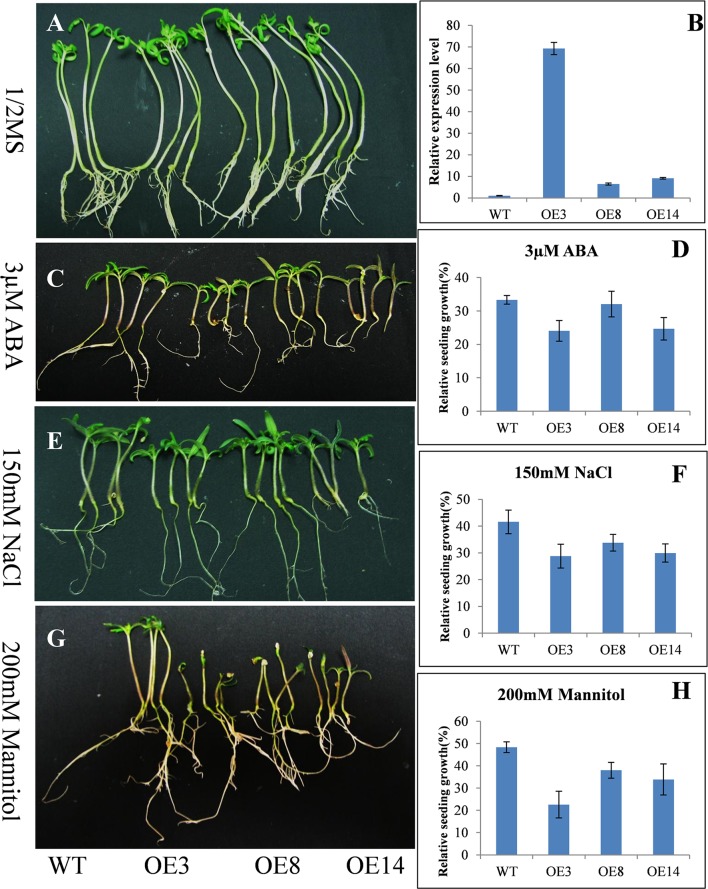
**Response of the tomato seedlings with overexpressed (OE) *HyPRP1* under stress conditions**. Growth of wild type (WT) and transgenic (OE3, OE8, and OE14) plants post-ABA, salt or mannitol treatments **(C,E,G)** compared with 1/2 MS-grown controls **(A)**. Each of four seedlings represents the line WT, OE3, OE8 and OE14 respectively. **(B)** Analysis of *HyPRP1* transcriptional expression via qRT-PCR in overexpressed (OE3, OE8, and OE14) and WT lines. **(D,F,H)** Significant differences in relative growth rates of OE and WT plants in ABA, salt, or mannitol treatment and without stress control conditions.

Real-time RT-PCR detection results showed that *SpHyPRP1* was highly expressed in tomato leaves. Surprisingly, *SpHyPRP1* expression was significantly suppressed by various abiotic stresses, including drought, high salinity, cold, heat, wounding, MV, and ABA (Figure 2). These expression patterns indicated that *SpHyPRP1* should be a negative regulator of abiotic stress and ABA. Additionally, *E. coli* cells with SpHyPRP1 were more sensitive to ROS. Thus, to further investigate the function of *HyPRP* in plant cells, the *SpHyPRP1* gene driven by 35S was introduced into cultivated tomato M82. Fifteen transformants (T_0_) were obtained, and three T_2_ homozygous lines with *HyPRP1* transcripts 69.3-fold (OE3), 6.4-fold (OE8), and 9.1-fold (OE14) greater than those of WT were screened out for further analysis (Figure 3B).

To test the abiotic-stress tolerance, transgenic seedlings overexpressing *SpHyPRP1* were grown on 1/2 MS media supplemented with 150 mM NaCl, 200 mM mannitol, or 3 μM ABA. No significant difference was observed between transgenic and WT seedlings in regular 1/2 MS media (Figure 3A). However, WT seedlings grew higher than transgenic lines in the media with NaCl, mannitol, or ABA (Figures 3C,E,G). For example, in NaCl treatment, the average plant heights of WT, OE3, OE8, and OE14 plants were 2.58, 1.66, 1.89, and 2.05 cm, respectively. Thus, OE3, OE8, and OE13 plants were 64.34, 73.25, and 79.46% shorter than WT, correspondingly. Compared with the seedlings in 1/2 MS media, the relative growth of transgenic seedlings significantly decreased in WT plants (Figures 3D,F,H). These results indicated that the transformants with overexpressed *SpHyPRP1* showed reduced resistance to salt, osmotic, and ABA stresses, suggesting that *SpHyPRP1* should be a negative regulator of abiotic stress in tomato.

### Knockdown of *SlHyPRP1* enhanced tomato tolerance to salt and oxidative stresses

**Figure 4:**
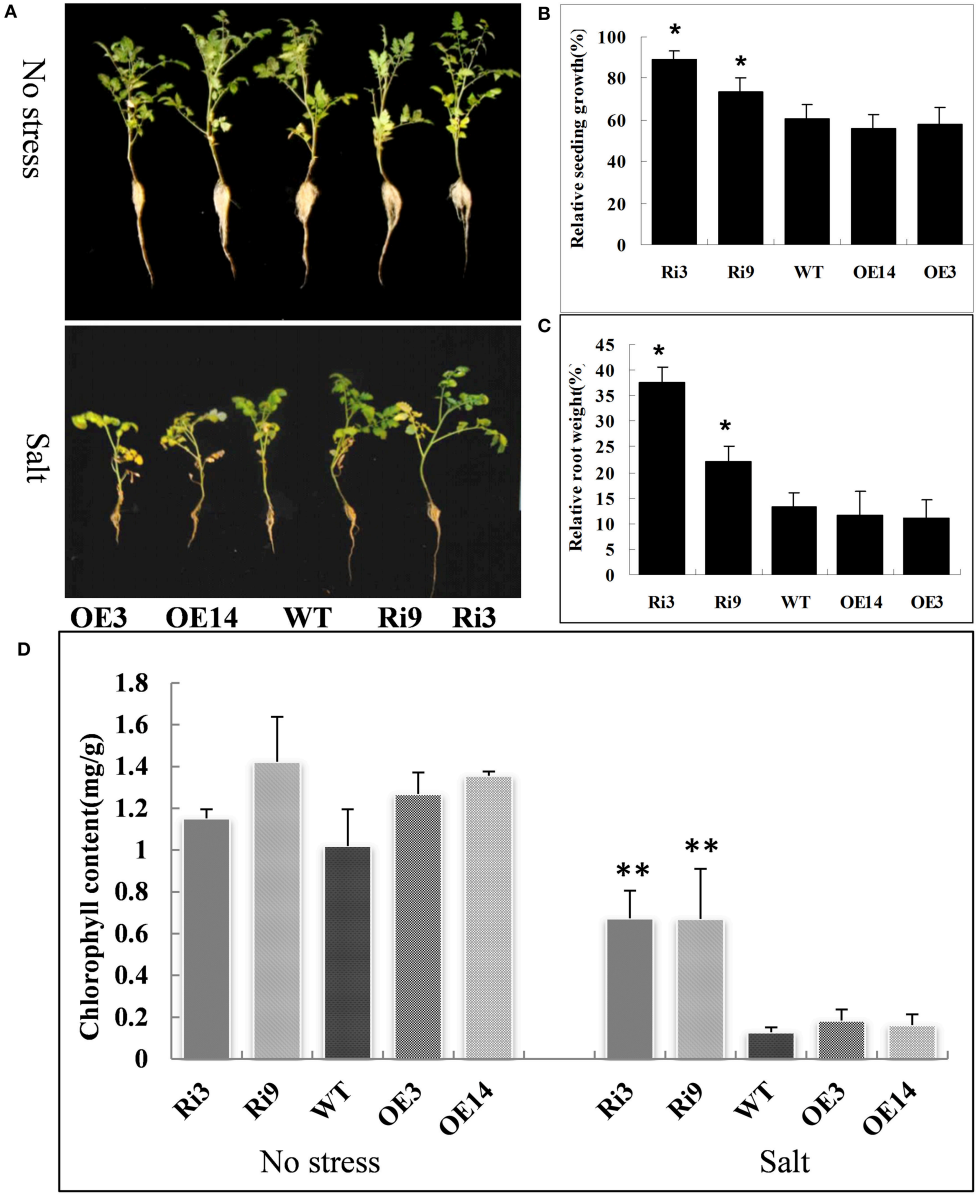
***SlHyPRP1*-RNAi (Ri) transgenic tomato plants have significantly improved salt stress tolerance compared with the wild type (WT) and *HyPRP1* overexpressed (OE) transgenic plants**. **(A)** Growth of transgenic and WT plants under non-stress (upper panel) and salt stress (bottom panel) in the field. **(B,C)** Significant differences in relative growth rates along transgenic and WT lines under salt treatment and no stress control conditions, respectively. Error bars indicate ± SE of the means (*) significant difference at *P* < 0.05. **(D)** The chlorophyll content of salt-treated and untreated leaves from wild-type (WT) and RNAi (Ri) plants. Error bars indicate ± SE of the means and (**) indicates significant difference at *P* < 0.01.

**Figure 5:**
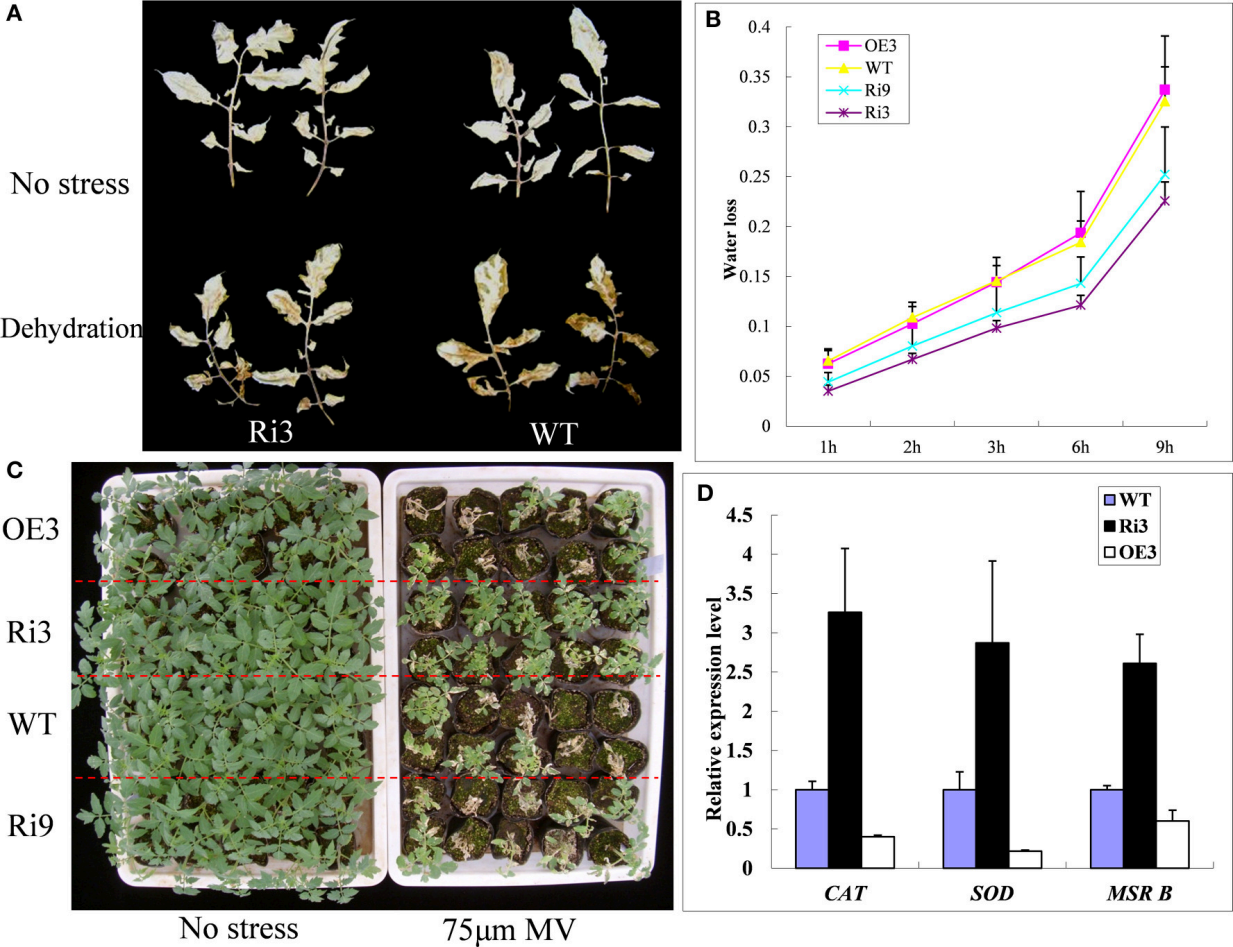
***SlHyPRP1*-RNAi (Ri) transgenic plants have improved oxidative stress tolerance. (A)** Accumulation of H_2_O_2_ in the WT and transgenic lines (Ri-3) under dehydration measured by histochemical staining with DAB. The leaves of WT and Ri-3 before detached (upper panel) and after detached for 3 h (lower panel). **(B)** Water loss rate of the transgenic and WT plants expressed as a percentage of the initial fresh weight (*n* = 15). **(C)** Comparison of the growth of T_2_ transgenic tomato plants post-treatment of oxidative stress to the non-stress control. **(D)** The transcript levels of super oxygen dehydrogenases (SOD), catalase (CAT), and methionine sulfoxide reductase (MSR B) were quantitatively analyzed in WT and *SlHyPRP1*-RNAi transgenic lines. Error bars indicate ± SE of means (*n* = 3).

To further analyze the *HyPRP1*, we suppressed its expression in *S. lycopersicum* cv. M82 by using RNAi. A total of 21 transformants (T_0_) were obtained from kanamycin-resistant calli. The expression levels were significantly knocked down in *SlHyPRP1*-RNAi transgenic T_2_ lines (Ri3: 0.02-fold and Ri9: 0.07-fold downregulated compared with WT) were obtained and used for the following abiotic stress analysis. At high salinity (200 mM NaCl), the growth rate and root weight of the seedlings of *SlHyPRP1*-RNAi transgenic lines were significantly higher than those of the WT control seedlings; the plants that exhibited overexpression showed slightly lower growth rate and root weight than those of the WT control seedlings, but no significant differences were observed (Figures 4A–C). Although no obvious difference was observed under non-stress conditions, the significantly higher chlorophyll contents were retained in *SlHyPRP1*-RNAi plants than in WT plants under the high-salinity treatment (Figure 4D). These results suggested that the knockdown of *SlHyPRP1* can enhance salt tolerance in tomato. In addition, water loss from detached leaves of WT occurred more rapidly than in the *SlHyPRP1*-RNAi transgenic lines (Figure 5B), and histochemical staining by DAB revealed that the accumulation of H_2_O_2_ *in situ* was less intense in *SlHyPRP1*-RNAi leaves after 3 h of dehydration (Figure 5A). These results suggest that the knockdown of *HyPRP1* enhanced salt and dehydration tolerance in tomato by scavenging ROS-like H_2_O_2_, which correspondingly improved the oxidative tolerance.

To further determine whether the knockdown of *SlHyPRP1* enhanced the oxidative tolerance of tomato, we used MV treatment to induce a membrane-lipid peroxidation leading to oxidative stress (Tsugane et al., 1999). After 3–4 days of treatment with 75 μM MV, the plant survival rates of Ri3 and Ri9 were 93 and 76%, respectively; only 43 and 40% were observed on the WT and OE transgenic lines (Figure 5C). To determine whether silencing of *SlHyPRP1* increased the expression of antioxidant-related genes, the expression patterns of these genes in *SlHyPRP1*-RNAi transgenic lines were analyzed. The transcripts of the tested ROS-scavenging genes (SOD, CAT, and Msr B) were increased twofold to fourfold in *SlHyPRP1*-RNAi transgenic plants compared with WT plants, whereas all genes were downregulated in the *HyPRP1*-overexpressed transgenic lines (Figure 5D). These results suggest that downregulation of *HyPRP1* would enhance tomato tolerance to salt and oxidative stresses by modulating the expression of ROS-scavenging genes.

### HyPRP1 interacts with Msr A, SO, Fds, and UBQ10 proteins

**Figure 6:**
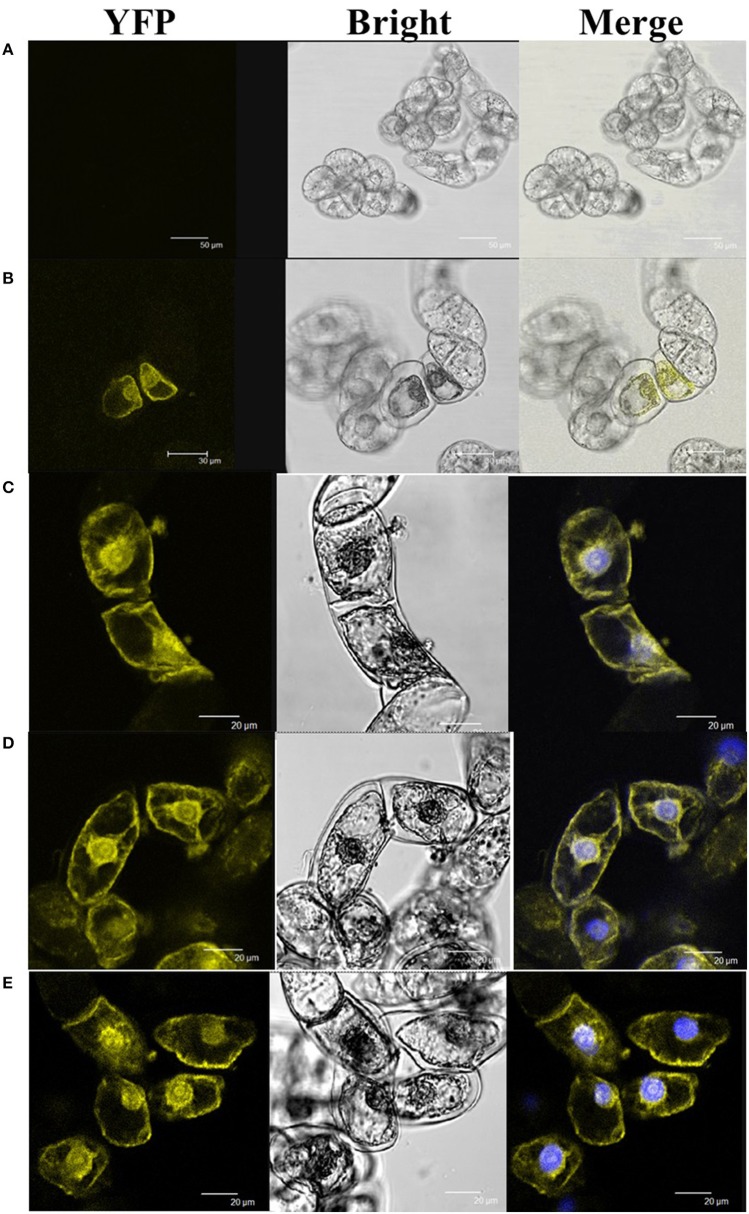
**BiFC visualization of the target interaction partners in cv BY-2 tobacco cells**. The counterpart proteins were tested in a pairwise fashion by fusing the full-length genes separately to each of the N- and C-terminal fragments of YFP. Each co-transformed with **(A)** HyPRP1::YFP^N^+YFP^C^, **(B)** HyPRP1::YFP^N^+SO::YFP^C^, **(C)** HyPRP1:: YFP^N^ + Msr:: YFP^C^, **(D)** HyPRP1:: YFP^N^ + Fd:: YFP^C^, and **(E)** HyPRP1:: YFP^N^ + UBQ:: YFP^C^. The photographs were taken under bright light (Bright), in a dark field for YFP-derived fluorescence (YFP), and merged, are presented.

To reveal the molecular mechanism of *HyPRP1* in plant response to abiotic stress, Y2H screening was performed to identify the HyPRP1-interacting proteins. By using HyPRP1 as bait and the cDNA library of tomato as prey, five relative proteins, namely, Msr A (methionine sulfoxide reductase A: P54153.1), UBQ10 (polyubiquitin: SGN-U580864), Fds (ferredoxins: Q43517), ZPR1 (ZPR1-type zinc finger protein: SGN-U576075), and SO (sulfite oxidase: ABI53846.1), were screened out. BiFC experiments were then performed to confirm the interactions. Pairwise expression of HyPRP1::YFP^N^ with UBQ10::SPYCE^C^, Msr A::SPYCE^C^, Fds::SPYCE^C^, SO::SPYCE^C^, or ZPR1::SPYCE^C^ all resulted in the accumulation of YFP fluorescence in the transformed BY2 cells, whereas no YFP fluorescence was observed in the control cells (Figure 6). These results clearly showed that the HyPRP1 protein can interact with Msr A, SO, UBQ10, Fds, and ZPR1 in plant cells.

The HyPRP1 interactive proteins were further analyzed under various abiotic stresses and plant growth regulator treatments. In the HyPRP1-interacting protein Msr A with 196 amino acids, the corresponding gene *Msr A* was strongly induced by exogenous ethylene and oxidative stress (100 μM MV). At 12 h after stress treatment, the transcripts increased more than 10-fold (Figure 1S). This finding implied that *Msr A* is a downstream factor in the abiotic-stress response. The transcription of *Fds* was suppressed after 12 h of exposure to drought and oxidative stress. The expression pattern of *SO* was similar to that of *Fds* (Figure 1S). These results indicated that the HyPRP1-interacting proteins respond to abiotic stresses and revealed the molecular mechanism underlying the oxidative stress tolerance of silenced *HyPRP1* in tomato.

### *HyPRP1* involved in sulfite metabolism

**Figure 7:**
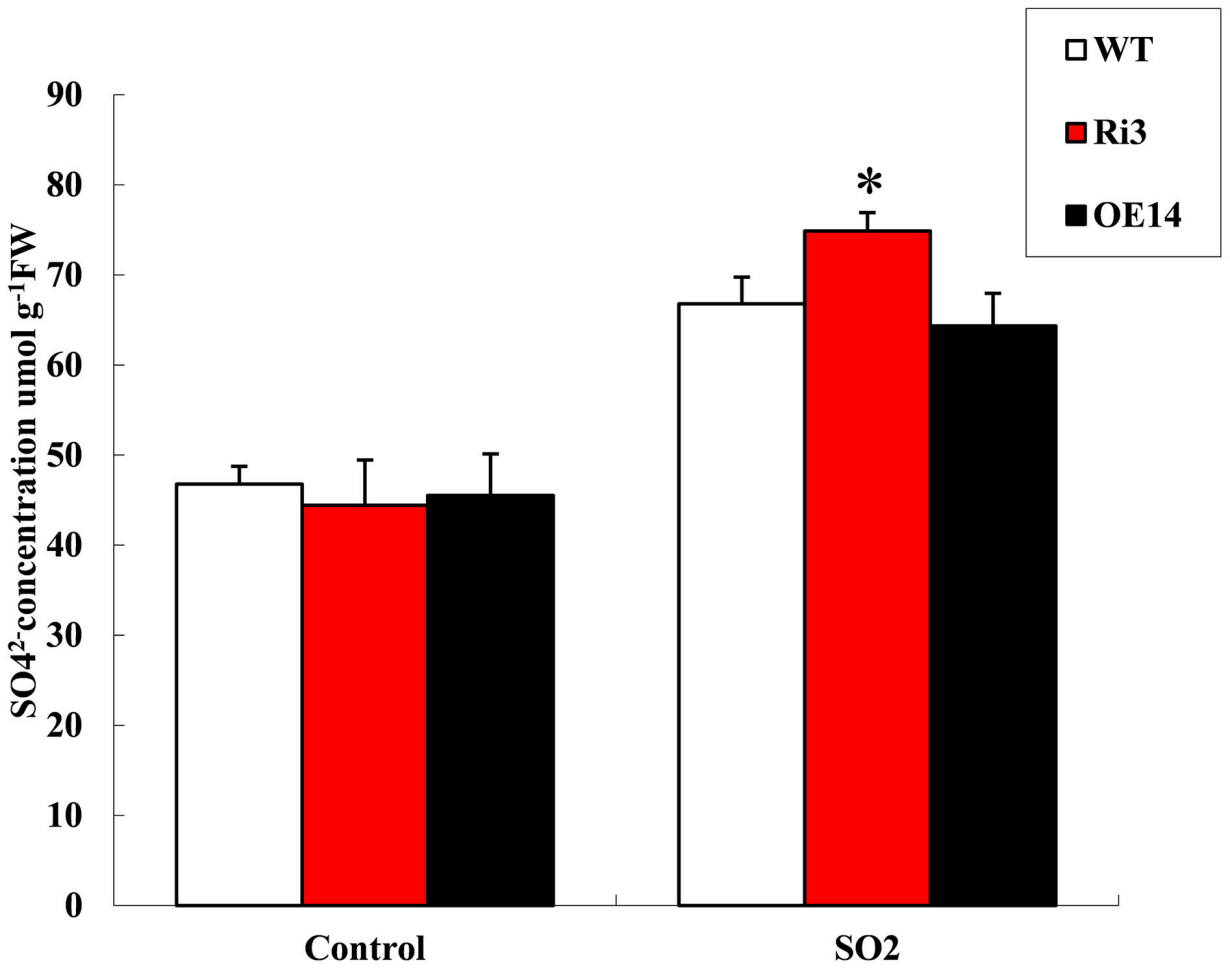
**Sulfate concentration under 10 ppm SO_2_ treatment for 2 h and untreated leaves from Ri, overexpressed, and WT plants**. Data are presented as means ± SE from three technical replications (μmol/g fresh weight). The difference between Ri and WT leaves indicated ^*^P < 0.05.

SO and Fds can detoxify sulfite (Leustek et al., 2000; Hansch and Mendel, 2005; Brychkova et al., 2007); hence, they act as sulfite antioxidant enzymes and donors for sulfite reductase. To determine whether *HyPRP1* is involved in sulfite metabolism, the sulfate contents of transgenic lines were measured before and after SO_2_ treatment. The results showed that SO_2_ can readily react with water to form sulfite, which adversely affects plant health. Instead of sulfite content, the sulfate concentration was monitored because sulfite levels are very low in plant tissues and are rapidly oxidized in extracts (Tsakraklides et al., 2002). To differentiate the sulfate contents of *HyPRP1* knockdown, overexpression lines and WT control plants after 2 h of SO_2_ treatment were exposed to 10 ppm SO_2_. The leaves corresponding to all the transgenic lines accumulated high concentrations of sulfate after SO_2_ treatment. The sulfate content was 30.5% in *SlHyPRP1*-RNAi lines, whereas it was only 20.0% and 18.8% in WT and overexpressing plants, respectively (Figure 7). These results indicated that *SlHyPRP1*-RNAi transgenic plants can catalyze the conversion of sulfite to non-toxic sulfate when the plants are subjected to SO_2_ pollution.

### *SlHyPRP1* knockdown lines can accumulate more transcripts of *Msr A* and *Fds* than wild-type plants under SO_2_ stress

**Figure 8:**
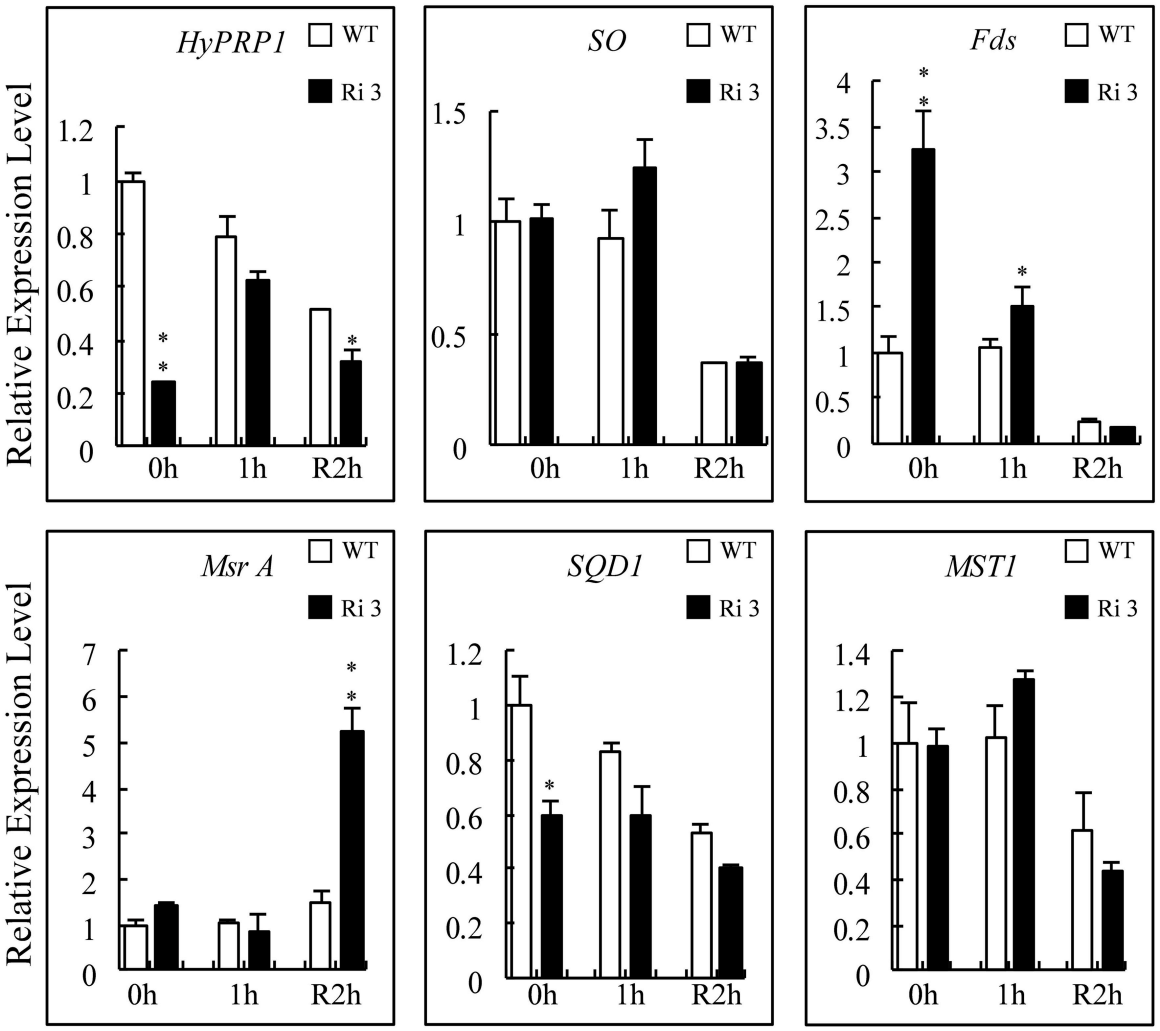
**Expression levels of HyPRP1-interacting genes and tomato sulfite-utilizing genes after SO_2_ exposure**. The expression levels of *SO, Fds, Msr A, MST1*, and *SQD1* were monitored by qRT-PCR analysis using wild-type (WT) and *SlHyPRP1*-RNAi (Ri) plants sampled immediately after 1 h of 4 ppm SO_2_ exposure (1 h) and later recovered for 2 h. All samples were collected at the indicated time points from three biological replicates of each treatment. Error bars indicate ± SE of the means (*n* = 3). The difference between Ri and WT indicated ^*^P < 0.05, ^**^P < 0.01.

Compared with the sulfite-reduced pathway, *SQD1* (sulfolipid biosynthesis protein; SGN-U217001), and *MST1* (Thiosulfate sulfurtransferase; SGN-U320318) are late-responsive (24 h) SO-dependent upregulated genes that catalyze the diversion of sulfite to other assimilatory pathways (Brychkova et al., 2007). The Msr A, SO, and Fds proteins were found to interact with HyPRP1. Thus, the transcript levels of their corresponding genes in WT and *SlHyPRP1*-RNAi plants exposed to 4 ppm SO_2_ for 1 h were monitored. The results showed that the transcript levels of *SO* and *MST1* were not distinctly changed in WT and *SlHyPRP1*-RNAi plants before and after exposure to SO_2_ or subsequent 2 h recovery (Figure 8). By contrast, the transcripts of *Fds* and *Msr A* significantly increased after exposure to SO_2_ for 1 h and subsequent 2 h recovery in *SlHyPRP1*-RNAi plants, respectively. However, *SQD1* was always downregulated before and after SO_2_ toxicity or subsequent 2 h recovery in *SlHyPRP1*-RNAi lines (Figure 8). These results indicated that the expression patterns of *Msr A, Fds*, and *SQD1* changed in *HyPRP1*-suppressed lines when exposed to SO_2_ toxicity.

## Discussion

### SpHyPRP1 and SlHyPRP1 share different structural features

*SpHyPRP1* and *SlHyPRP1* showed different ROS-scavenging ability in *E. coli* cells, which should be attributed to their individual amino acid sequences and protein structures. Both SpHyPRP1 and SlHyPRP1 contain the same PRD and 8CM domains, and variations in eight amino acids were shown within a predicted transmembrane domain (Figure 1A). Of the two variant residues, the average hydrophobicity of SlHyPRP1 was higher than that of SpHyPRP1, and the amino acid 96 of SlHyPRP1 (Ile, a hydrophobic amino acid) was replaced by Asn (a hydrophilic one) in SpHyPRP1. These changes may affect the protein–protein interaction and binding site for a lipid or lipid-soluble effector molecule(s) of the protein (Ma et al., 2009).

Abiotic stresses can alter the expression of responsive genes by modulating DNA/protein methylation or demethylation in plants (Choi and Sano, 2007; Luo et al., 2012). The amino acid demethylation in *S*. *pennellii* LA0716 (Figure 1A) may play an essential role in regulating protein structure and functions related to abiotic stresses. However, the involvement of methylation or demethylation in abiotic stress response remains unclear; the resulting amino acid variations may contribute to the ecotype adaptation of tomato. Although, our results demonstrated that the ectopic expression of SlHyPRP1 and SpHyPRP1 in *E. coli* cells led to phenotypic differences under oxidative stress (Figures 1B,C), the significance of these changes in chronic plant domestication processes remains unexplained.

### *HyPRP1* is a novel negative regulator of ABA and abiotic stress and regulates the expression of antioxidant genes

Previous reports have shown that the expression of HyPRP genes was sensitive to environmental stimuli, such as pathogen infection, wounding, and drought (Goodwin et al., 1996; Gyorgyey et al., 1997; Otte and Barz, 2000). These findings are consistent with our observation that *SpHyPRP1* transcripts decreased rapidly in response to ABA and abiotic stress (Figure 2). Silencing of the negative regulator *HyPRP1* can induce the expression of antioxidant genes, such as that encoding superoxide dismutase and catalase, which enhanced oxidative tolerance (Figure 5). Conversely, in *HyPRP1*-overexpressing plants, the expression levels of the same ROS-scavenging genes were downregulated (Figure 5D). These findings are consistent with the case of *HyPRP1* in *N. benthamiana*, which serves as a negative regulator of basal defense against pathogens by negatively regulating the expression of defense-related and antioxidant genes (Yeom et al., 2012). However, the plants did not show significantly sensitive phenotypes in field conditions (Figures 4A, 5C) probably because an abiotic-sensitive species (M82) was selected for transformation (Gong et al., 2010) and more difficult to identify difference of drought tolerance in the field condition when the seedling grow up.

### Possible response mechanism of *HyPRP1* to abiotic stress

In most cases, the suppression of a negative regulator or the enhancement of a positive regulator of ABA would appear to confer drought tolerance (Oh et al., 2005; Pandey et al., 2005; Zhang et al., 2007). As a negative regulator of ABA, the suppression of *HyPRP1* can also significantly improve the seedlings' tolerance to salt stress in *S. lycopersicum* cv. M82 (Figure 4A) and enhance the seedlings' sensitivity to salt and mannitol in *HyPRP1*-overexpressed transgenic tomato lines (Figure 3). Leaves detached from *SlHyPRP1*-RNAi transgenic lines showed lower rates of water loss and less H_2_O_2_ accumulation than those from the WT (Figures 5A,B). Moreover, HyPRP1 can bind with the Msr A protein, which is a type of oxidoreductase (Doney and Thompson, 1966) responding to oxidative stress (Figure 1S). Together with the expression of Msr B, HyPRP1 is higher in *SlHyPRP1*-RNAi plants but lower in *HyPRP1*-overexpressed lines (Figure 5D). Overall, the results illustrated that *HyPRP1* might affect ROS scavenging by binding with few oxidoreductases, and these binds can suppress the activities of those oxidoreductases. The removal of the Msr enzyme in mammals can lead to the loss of their antioxidant defense, resulting in enhanced oxidative damage and decreased lifespans (Moskovitz et al., 2001). Msr B can actively defend against pathogens by regulating the cell redox status and reducing the production of ROS (Oh et al., 2010). This finding provides another evidence indicating that *HyPRP1* can negatively regulate the *Msr* genes.

HyPRP1 also bound with ZPR1 protein, which is involved in the ABA signaling network and plays a potential role in plant cell development and abiotic stress response in tomato (Li et al., 2013). ZPR1 was initially identified in mammals as a cytoplasmic zinc finger protein, which is essential for cell viability and normal cellular proliferation (Gangwani et al., 1998). However, the participation of ZPR1 in signaling of abiotic responses and the effect of its interaction with protein HyPRP1 should be clarified. Ubiquitin (UBQ) is a small regulatory protein found in almost all tissues of eukaryotic organisms, which binds to proteins and labels them for degradation through the UBQ–proteasome pathway (Hochstrasser, 2009; Kimura and Tanaka, 2010). HyPRP1 interaction with UBQ can help us understand the UBQ–proteasome pathway under abiotic stresses. However, the exact role of ubiquitination in abiotic responses has not been elucidated in higher plants.

HyPRP1 interacted with several abiotic-response genes, suggesting that it acts along a signaling pathway and not as a final component. *HyPRP1* modulated *Msr A, SO, ZPR1*, and *Fds* to confer tolerance to oxidative and salt stresses. However, the mechanism by which these proteins cooperate or co-regulate with each other at the transcriptional, post-transcriptional, or protein levels remains undetermined. Exploration of the detailed mechanism of how UBQ binds with HyPRP1 and how ubiquitination is induced by abiotic stresses will be of significant interest.

### *HyPRP1* is involved in sulfite metabolism

SO_2_ is an external source of toxic stimuli for plants and can react with water to form sulfite, which causes direct damage to plants by turning their leaves yellow and bleaching them upon entering the stomata (Brychkova et al., 2007; Lang et al., 2007). Air pollution caused by SO_2_ results in acid rain, which causes direct visible oxidative damage to plant tissues (Vickers et al., 2009), including chlorophyll destruction, death of plant tissue, and long-term yield reduction (Noji et al., 2001; Kong et al., 2002). SO (EC 1.8.3.1) is believed to be required to convert the extra oxidized sulfite back to sulfate when plants are subjected to SO_2_ gas (Hansch and Mendel, 2005). Overexpression of the *SlSO* gene in tomato and *A. thaliana* can catalyze the transformation of sulfites into non-toxic sulfate and protect plants against SO_2_ toxicity; by monitoring sulfate concentrations before and after fumigation by SO_2_, more sulfite was detected and converted to sulfate in WT plants than in *SO* knockdown plants (Brychkova et al., 2007). In the current study, more sulfite was converted to sulfate in *SlHyPRP1*-RNAi transgenic plants than in *HyPRP1*-overexpressed and WT plants after SO_2_ treatment (Figure 7). These results implied that the absence of *HyPRP1* may improve SO activity when plants are exposed to SO_2_ phytotoxicity. However, the overexpression of *HyPRP1* cannot impair the function of SO because the sulfate content does not significantly increase in *HyPRP1*-overexpressed plants compared with WT plants. Conceivably, the overexpression of *SpHyPRP1* partially inhibited SO, which is similar to the results in *E. coli* cells where the overexpressed *SpHyPRP1* only showed slightly reduced oxidative tolerance.

Fds acts as a physiological donor of six electrons required for sulfite reductase (SiR; EC 1.8.7.1), whereas SiR uses NADPH in bacteria (Yonekura-Sakakibara et al., 2000; Oh et al., 2005). The sulfite can also be reduced by SiR through a deoxidation process that transfers six electrons of Fds to produce hydrogen sulfide (Leustek et al., 2000). Our expression analysis results indicated that *SO* was not responsive to SO_2_ treatment. However, *Msr A* and *Fds* were significantly upregulated among the *HyPRP1* knockdown lines during SO_2_ treatment (Figure 8). The expression level of *SO* remained unchanged before and after SO_2_ treatment, which confirms that the *SO* transcript levels are not highly sensitive to SO_2_ application (Brychkova et al., 2007). Other sulfite-utilizing genes (Sanda et al., 2001; Tsakraklides et al., 2002) such as *SQD1* were downregulated after SO_2_ damage, and the *MST1* transcript was similar in both WT and *HyPRP1* knockdown transgenic lines (Figure 8). These results indicate that *HyPRP1* was involved in sulfite metabolism by binding with related enzymes and by regulating the expression of related genes.

## Author contributions

JZ and ZY conceived the study; JL and WS performed the experiments; TW and ZL analyzed the data; BO, CY, and HL provided the reagents and tools; JL and JZ wrote the paper. All the authors have discussed the results and contributed to improving the paper.

### Conflict of interest statement

The authors declare that the research was conducted in the absence of any commercial or financial relationships that could be construed as a potential conflict of interest.
