# Supplementary material for: HyPRP1 Gene Suppressed by Multiple Stresses Plays a Negative Role in Abiotic Stress Tolerance in Tomato
Source: Front Plant Sci. 2016 Jun 29;7:967. doi: 10.3389/fpls.2016.00967 (PMC4925714; doi:10.3389/fpls.2016.00967)
Supplement: Supplementary file 1 [file Table1.PDF]

Table 1S | Primer sequences used for real-time RT-PCR and BiFC analysis.

Primer		Sequence (5'-3')
HyPRP	qRT-PCR	Forward CCATCACCTAAAGGGAAAAAACC<br>Reverse GCCCACTAAGTATTGGACAACATTC
MSR A	qRT-PCR	Forward CAAGTCAACCACCAATCCA<br>Reverse TCACTACTCCTCCAACCCTC
Fds	qRT-PCR	Forward GGCTGGTTCTTGCTCATCTT<br>Reverse ATGGTAACATCACCCTTTGG
CAT	qRT-PCR	Forward AAGTCCTGTGGTCAGAAGGTCG<br>Reverse GAAGTACAGTTTATAGCACAACGCG
Msr B	qRT-PCR	Forward TGGCAGGAGGGTTGAGAT<br>Reverse GCTGGTGTAAACTTGAGGGA
SOD	qRT-PCR	Forward TGAATTGGGGTTGAACCATT<br>Reverse GCAGGCACTGTAATCTGCAA
SO	qRT-PCR	Forward ATTCCATTGAGTCAGGCTACA<br>Reverse CACACCAGGGACAACCACAC
MST1	qRT-PCR	Forward TCAAGAGTATCAGGTTGCACATATTCC<br>Reverse CTAAAGATTCCCTTCCCATCATAGACA
SQD1	qRT-PCR	Forward GTTGACAACCTTATCCGTCGATTATTT<br>Reverse GACTACAGCATCAGGTTCAAAGGATTT
β-Actin	qRT-PCR	Forward GTCCTCTTCCAGCCATCCAT<br>Reverse ACCACTGAGCACAATGTTACCG
HyPRP	BiFC	Forward <u>GTCGAC</u> ATGGAGTTCTCTAAGATAACTTC<br>Reverse <u>GTCGAC GGTACC</u> GATGGAACAAGTGTAGCCAGG
MSR A	BiFC	Forward <u>GTCGAC</u> ATGGGGAGTAACAGCAGCAGC<br>Reverse <u>GTCGAC GGTACC</u> ACCGTAGCACTTTATTGGATC
SO	BiFC	Forward <u>GTCGAC</u> ATGCCTGGGATTAAAGGGCCT<br>Reverse <u>GTCGAC GGTACC</u> AAGATTTGCTTGACCAACTCGGA
Fds	BiFC	Forward <u>GTCGAC</u> ATGGCTAGTATTTCTGGTACAATG<br>Reverse <u>GTCGAC GGTACC</u> AGCAGTAAGCTCCTCCTCCTTGTG
UBQ	BiFC	Forward GC<u>TCTAGA</u>ATGCAGATCTTTGTGAAAACTCTCA<br>Reverse GC<u>TCTAGA</u>GGTACCAAAACCACCACGGAGACGG
