# Supplementary material for: HyPRP1 Gene Suppressed by Multiple Stresses Plays a Negative Role in Abiotic Stress Tolerance in Tomato
Source: Front Plant Sci. 2016 Jun 29;7:967. doi: 10.3389/fpls.2016.00967 (PMC4925714; doi:10.3389/fpls.2016.00967)

Figure 1S | Expression patterns of *Msr A*, *Fds*, and *SO* under various abiotic stresses(e.g., drought, salt, heat, cold, and MV) and ABA treatments.

The leaves of tomato plants under various stresses (drought, salt, wounding, heat, cold, MV) and plant growth regulator treatments (100 μM ABA, 1 mM ethylene, and 100 μM GA_3) were sampled at designed time points. All samples were collected at indicated time points from three biological replicates of each treatment. Error bars indicate ± SE of means (n=3).

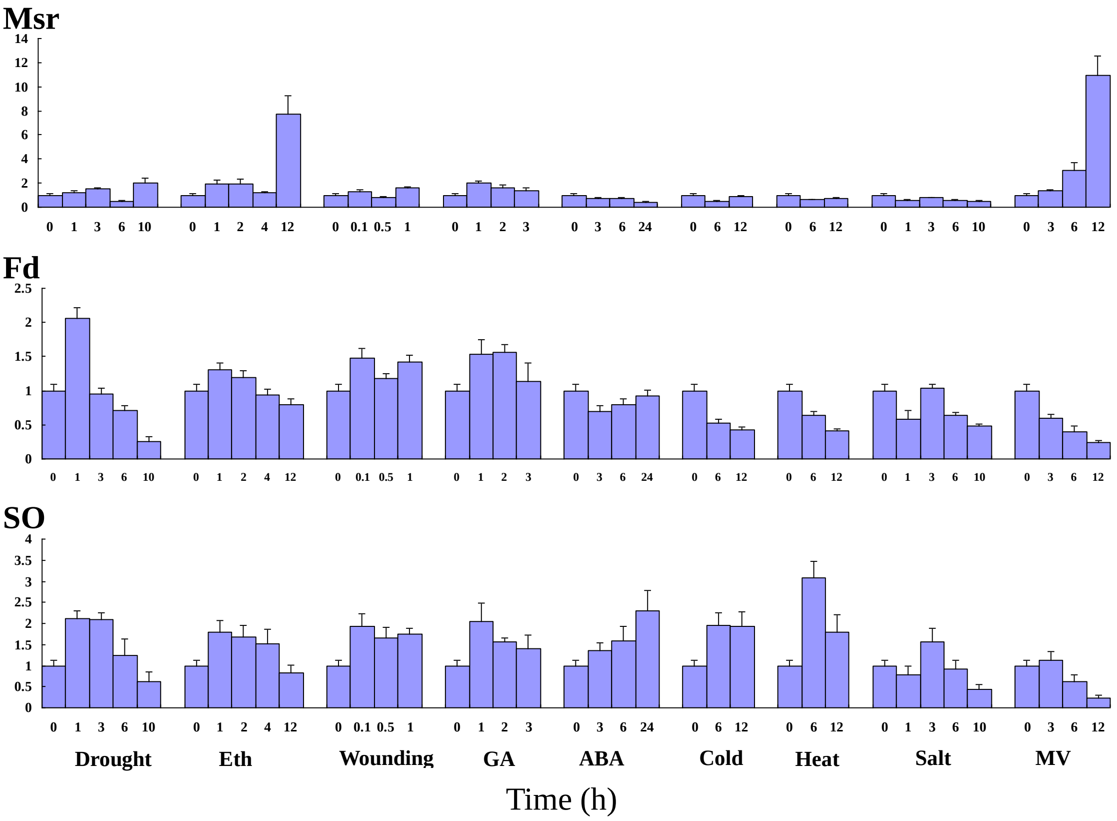

Supplement: Supplementary file 2 [file Image1.PDF]
